# PGC-1β-expressing POMC neurons mediate the effect of leptin on thermoregulation in the mouse

**DOI:** 10.1038/s41598-020-73794-7

**Published:** 2020-10-15

**Authors:** Julien Delezie, Jonathan F. Gill, Gesa Santos, Bettina Karrer-Cardel, Christoph Handschin

**Affiliations:** Biozentrum, University of Basel, Klingelbergstrasse 50/70, 4056 Basel, Switzerland

**Keywords:** Hypothalamus, Mitochondria, Transcription, Hypothalamus, Metabolism, Obesity

## Abstract

The arcuate nucleus (ARC) of the hypothalamus is a key regulator of food intake, brown adipose tissue (BAT) thermogenesis, and locomotor activity. Whole-body deficiency of the transcriptional coactivator peroxisome proliferator-activated receptor γ (PPARγ) coactivator-1β (PGC-1β) disrupts mouse circadian locomotor activity and BAT-regulated thermogenesis, in association with altered gene expression at the central level. We examined whether PGC-1β expression in the ARC is required for proper energy balance and locomotor behavior by generating mice lacking the PGC-1β gene specifically in pro-opiomelanocortin (POMC) neurons. POMC neuron-specific deletion of PGC-1β did not impact locomotor behavior, food intake, body composition, energy fuel utilization and metabolic rate in fed, 24-h fasted and 24-h refed conditions. In contrast, in the fed state, deletion of PGC-1β in POMC cells elevated core body temperature during the nighttime period. Importantly, this higher body temperature is not associated with changes in BAT function and gene expression. Conversely, we provide evidence that mice lacking PGC-1β in POMC neurons are more sensitive to the effect of leptin on heat dissipation. Our data indicate that PGC-1β-expressing POMC neurons are part of a circuit controlling body temperature homeostasis and that PGC-1β function in these neurons is involved in the thermoregulatory effect of leptin.

## Introduction

The arcuate nucleus (ARC) of the hypothalamus contains pro-opiomelanocortin (POMC)- and agouti-related peptide (AgRP)-expressing neurons that respond to bidirectional changes in energy availability and major metabolic hormones such as leptin^1–3^. Besides integrating multiple inputs, the ARC sends widespread projections to different brain areas including e.g., the lateral hypothalamus, the paraventricular nucleus of the hypothalamus, and the brainstem^4–8^. The ARC is therefore a key regulator of appetite and satiety, brown adipose tissue (BAT) thermogenesis, glucose metabolism, locomotor activity, and hormone secretion^9–13^.

Several key proteins and kinases expressed within mouse POMC and AgRP neurons are essential to coordinate energy balance^14^. For instance, targeted deletion of mitofusin-2 (MFN2)—a protein involved in mitochondrial dynamics—in mouse POMC neurons alters energy expenditure and BAT thermogenesis^15^. We have likewise demonstrated that specific deletion of the transcriptional coactivator peroxisome proliferator-activated receptor γ (PPARγ) coactivator-1α (PGC-1α) in AgRP neurons disrupts leptin sensitivity and food intake^16^. The role of the related PGC-1β in this process is unclear, even though this coactivator shares some overlapping function with PGC-1α in several tissues^17–19^. Like PGC-1α, PGC-1β is expressed at high levels in different mouse brain regions including the hypothalamus^20^. Accordingly, whole-body PGC-1β deletion alters the expression of a large number of nuclear-encoded genes governing mitochondrial and metabolic functions in hypothalamic cells^21^. Moreover, global PGC-1β deficiency disrupts circadian locomotor activity and BAT-regulated thermogenesis^22,23^. Yet, whether PGC-1β expression in the ARC nucleus is essential for the regulation of behavior and energy balance in the mouse remains unknown.

To address this issue, we have generated and characterized mice with a targeted deletion of *Ppargc1b*, the PGC-1β gene, in POMC neurons. We show that specific loss of PGC-1β in mouse POMC cells significantly elevates core body temperature (BT) at night, in the absence of change in locomotor activity, energy expenditure and BAT thermogenesis. Furthermore, we provide evidence for a PGC-1β-dependent modulation of leptin signaling that is linked to thermoregulation. Collectively, our findings suggest that hypothalamic PGC-1β is part of a leptin-driven sensing mechanism involved in the regulation of heat conservation in the mouse.

## Results

### Systemic energy homeostasis in the fed and fasted state is unchanged by PGC-1β deletion in POMC neurons

**Figure 1:**
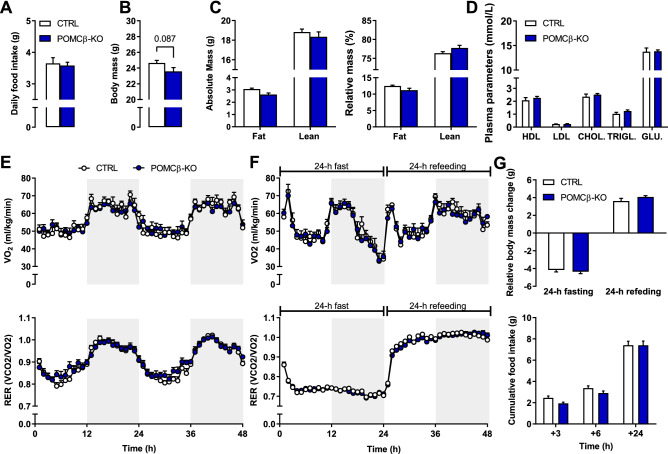
Unchanged energy homeostasis upon PGC-1β deletion in POMC neurons. (**A**) Daily food intake, (**B**) body mass and (**C**) absolute and normalized fat and lean mass of 3 to 4-month-old CTRL and POMCβ-KO mice. (**D**) Blood parameter analysis: high-density lipoprotein (HDL), low-density lipoprotein (LDL), cholesterol (CHOL.), triglycerides (TRIGL.) and glucose (GLU.) levels. (**E**) O_2_ consumption (*upper panel*) and respiratory exchange ratio (RER; *lower panel*) under basal fed conditions and (**F**) 24-h fasted-refed conditions with the grey background indicating night. (**G**) Relative body mass change under 24-h fasted-refed conditions (*upper panel*) and cumulated food intake during refeeding (*lower panel*). n = 7–8 per genotype for all measurements. Results are expressed as mean ± SEM. Unpaired Student's *t* test (**A**, **B**, **D**) and two-way ANOVA (**C**, **E**, **F**, **G**).

Deletion of PGC-1β in POMC neurons was achieved by crossing mice harboring a floxed *Ppargc1b* allele^17^ with mice that express Cre recombinase in the vast majority of POMC neurons^24^, hereafter called POMCβ-KO mice. Deletion of PGC-1β was restricted to the hypothalamus as determined by PCR analysis of the recombination event as well as qPCR evaluation of Cre expression in POMCβ-KO mice (Supplementary Figure 1A,B). Under ad libitum feeding conditions, daily food intake and body mass were comparable between littermate controls (CTRL; *Ppargc1b*^*flox/flox*^) and POMCβ-KO mice (Fig. 1A,B). Similarly, lean and fat mass were unchanged in POMCβ-KOs (Fig. 1C). Finally, daytime levels of plasma glucose and other circulating lipid-related parameters were not affected by PGC-1β deletion in POMC neurons (Fig. 1D).

We then used a comprehensive animal metabolic monitoring system (CLAMS) to evaluate systemic energy homeostasis in POMCβ-KO mice. In basal fed conditions, O_2_ consumption (VO_2_), energy expenditure and energy substrate utilization (respiratory exchange ratio; RER) were unaltered in POMCβ-KO mice (Fig. 1E; Supplementary Figure 1C). Moreover, temporal distribution of food intake was identical between genotypes (Supplementary Figure 1E). Likewise, 24-h fasting and refeeding experiments did not reveal any difference between POMCβ-KO and CTRL animals, as evidenced by unchanged VO_2,_ RER and heat production levels (Fig. 1F; Supplementary Figure 1D). Lastly, food intake and body mass changes were identical upon refeeding (Fig. 1G). Overall, these results show that food intake, adiposity, and energy expenditure are unchanged in the absence of PGC-1β in POMC cells.

### POMCβ-KO mice show elevated body temperature in the fed state in the absence of increased locomotor activity

**Figure 2:**
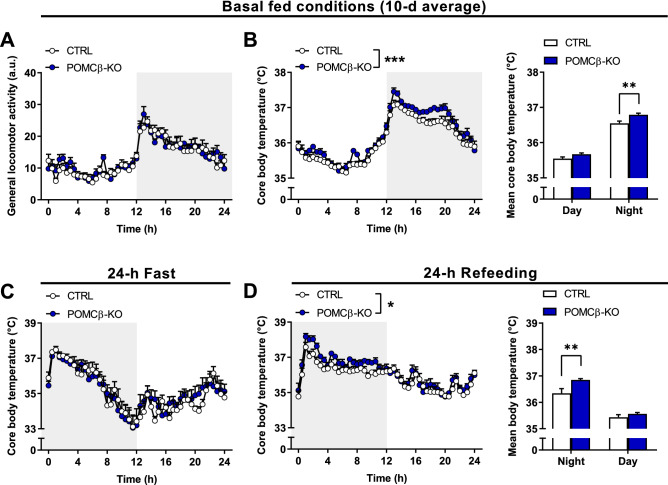
POMCβ-KO mice show elevated body temperature in the fed state. (**A**) Daily gross locomotor activity and (**B**) core body temperature levels of 3 to 4-month-old CTRL and POMCβ-KO mice in basal fed conditions as measured by telemetry over a 10-d period. (**C**) Core body temperature levels during a 24-h fasting period and (**D**) during a 24-h refeeding period with the grey background indicating night. n = 7–8 per genotype. Results are expressed as mean ± SEM. *P < 0.05; **P < 0.01; ***P < 0.001. Two-way ANOVA (**A**–**D**).

Whole-body PGC-1β deficiency disrupts locomotor activity levels and BAT-regulated thermogenesis^22^. To assess whether these changes originate from PGC-1β function in POMC neurons, we have monitored spontaneous locomotion and core BT over a 10-d period from single-caged mice housed in a ventilated cabinet—providing full control over external temperature (herein, 23 °C) and minimizing external disturbances. Incidentally, both mouse groups exhibited a similar body mass over prolonged single housing (Supplementary Figure 1F). Our telemetry results show that both daily patterns and levels of general locomotor activity were unaffected by PGC-1β deletion in basal fed conditions (Fig. 2A). Strikingly however, core BT was significantly elevated during the nighttime, active period in POMCβ-KO animals (Fig. 2B). To substantiate that the elevation of core BT was specific to PGC-1β deletion in POMC neurons, we have likewise evaluated mice lacking PGC-1β specifically in AgRP neurons in the exact same housing conditions (Supplementary Figure 2A). Besides unaltered body composition and food intake, these animals did not exhibit significant change in locomotor activity and BT levels as compared to their respective littermate controls (Supplementary Figure 2B–D).

In light of the absence of activity-driven thermogenesis, we next assessed whether the elevation of BT at night in POMCβ-KO animals was triggered by feeding. Thus, mice were fasted for 24 h (from early nighttime) and then subjected to refeeding for 24 h. Upon fasting, BT values in POMCβ-KO mice normalized to those of CTRL mice in the early nighttime and equally dropped to reach a minimum of ca. 33 °C in the late night, i.e. after 12 h of food deprivation (Fig. 2C). Likewise, prolonged fasting to the daytime period did not further reveal significant changes in BT in POMCβ-KO (Fig. 2C). In contrast, POMCβ-KO mice displayed higher BT levels upon refeeding (Fig. 2D), again in the absence of significant differences in locomotor activity and food intake (Supplementary Figure 2E,F). Lastly, the defective thermoregulation of POMCβ-KOs could not be observed when mice where housed at thermoneutrality (Supplementary Figure 2G,H).

Altogether, these experiments suggest that the elevated core BT of POMCβ-KO mice is associated with a thermic effect of food under sub-thermoneutral conditions.

### Loss of PGC-1β in POMC neurons does not alter BAT gene expression and thermogenic capacity

**Figure 3:**
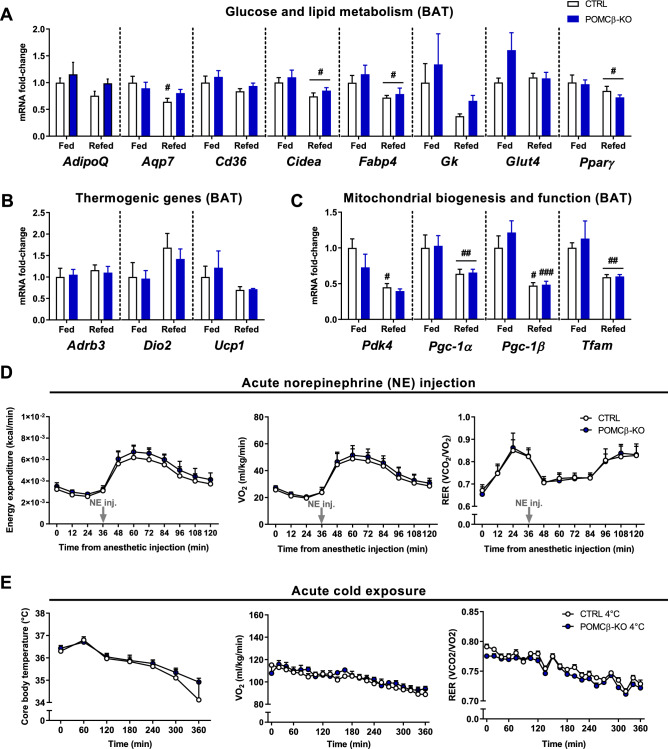
Loss of POMC-PGC-1β does not alter BAT gene expression and thermogenic capacity. (**A**–**C**) Gene expression in the brown-adipose tissue (BAT) of CTRL and POMCβ-KO mice. Expression values were determined by qPCR and normalized to *Actb*. Data is shown as the average fold-change ± SEM relative to the expression in CTRL (n = 4–5 per genotype). (**D**) Energy expenditure (*left*), O_2_ consumption (*middle*) and respiratory exchange ratio (RER; *right panel*) values following an acute injection of norepinephrine (NE inj.; 1 mg kg^−1^ i.p.) in anesthetized animals (n = 6–8 per genotype). (**E**) Core body temperature (*left*), O_2_ consumption (*middle*) and RER values (*right panel*) during acute cold exposure (n = 7–8 per genotype). ^#^Indicates a significant difference between experimental conditions for a given genotype (^#^*P* < 0.05; ^##^*P* < 0.01; ^###^*P* < 0.001). Two-way ANOVA (**A**–**E**).

BAT is a key regulator of mammalian thermogenesis, in particular during cold exposure and in the postprandial state^25^. Moreover, white adipose tissue (WAT) can acquire BAT characteristics in a process called beigeing/browning, and thereby also contribute to heat production in various contexts^26,27^. We thus evaluated the expression of metabolic genes and related markers of activation of BAT and beigeing of inguinal WAT of fed animals (sacrificed 3 h after the onset of their active/feeding period) and of mice fasted for 24 h and refed for 3 h in early nighttime (i.e., time period in which the difference in BT between genotypes is at its highest; see Fig. 2B,D). Our results show that the expression of genes mediating the uptake of energy fuel to sustain meal-induced thermogenesis in brown adipose cells^28^ were similarly changed across the different feeding conditions in both genotypes (Fig. 3A). Moreover, we did not notice any significant difference in the expression of BAT-specific (e.g., *Ucp-1*) and mitochondrial genes (e.g., *Pgc-1α*) between genotypes (BAT, Fig. 3B,C; iWAT, Supp Fig. 3A-C). Hence, the higher BT levels observed in POMCβ-KO mice do not correlate with substantial change in the abundance of major metabolic, pro-thermogenic or beige-enriched transcripts in BAT or WAT.

Expression of thermogenic genes (e.g., *Ucp1*) do not always correlate with BAT thermogenic capacity, especially at sub-thermoneutral temperatures^27,29,30^. In order to evaluate the BAT-specific thermogenic capacity, mice were injected with a supramaximal dose of norepinephrine (NE)—a broad adrenergic receptors agonist^31,32^. Importantly, animals were pre-exposed to thermoneutrality for 3-weeks in order to minimize BAT thermogenic activity^31^. Our results show that NE-induced changes in metabolic rate and energy fuel utilization (i.e., RER) were unaffected in the absence of PGC-1β in POMC neurons (Fig. 3D). Lastly, we evaluated the physiological responses of POMCβ-KOs housed at 23 °C to acute cold exposure—which mainly initiates shivering thermogenesis^31,32^. Cold-exposed POMCβ-KO and CTRL animals displayed a similar drop in BT and a similar elevation of their metabolic rates (Fig. 3E; Supp Fig. 3D,E). Collectively, these results indicate that both BAT and WAT, and to a lesser extent shivering, do not seem to contribute to the higher BT levels seen in POMCβ-KOs, in line with the unaltered metabolic rate of these mice. These findings are different from those obtained in global PGC-1β knockout mice, which are cold sensitive^22,23^.

### Mice lacking PGC-1β in POMC neurons are more sensitive to the thermoregulatory effects of leptin

**Figure 4:**
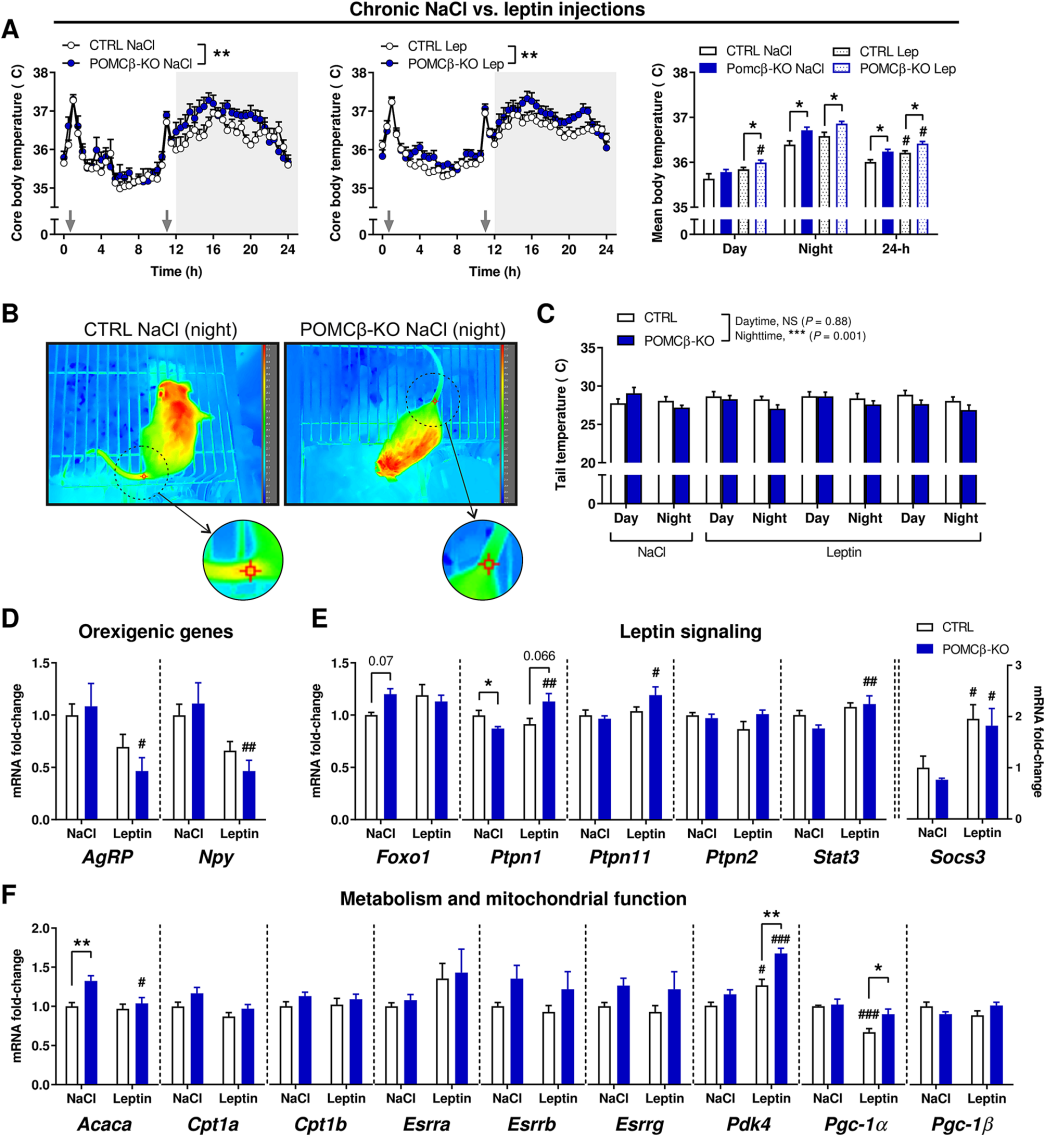
POMCβ-KO mice are more sensitive to the thermoregulatory effects of leptin. (**A**) 24-h core body temperature data of CTRL and POMCβ-KO mice in response to i.p. injections, twice daily, of NaCl (*left*), leptin (*middle*), and resulting average of day versus night and 24-h body temperature values (*right panel*). Gray arrows indicate the time of injection and grey background indicates the night period. (n = 7–8 per genotype). (**B**) Representative infrared thermography images of a CTRL and a POMCβ-KO mouse. The 3*3 block of pixels square indicate the region of interest from the tail base. (**C**) Tail temperature analysis from thermography images acquired during day and night from CTRL and POMCβ-KO mice treated with either saline or leptin. (**D**–**F**) Gene expression in the arcuate nucleus of CTRL and POMCβ-KO mice. Expression values were determined by qPCR and normalized to *Hprt*. Data is shown as the average fold-change ± SEM relative to the expression in CTRL (n = 4–7 per genotype per condition). *Indicates a significant difference between genotypes (*P < 0.05; **P < 0.01; ***P < 0.001); ^#^indicates a significant difference between experimental conditions for a given genotype (^#^*P* < 0.05; ^##^*P* < 0.01; ^###^*P* < 0.001). Unpaired Student's *t* test (**A** [right panel]) and two-way ANOVA (**A** [middle and right panels], **B**–**E**).

Insulin and leptin are essential hormones controlling food intake and energy expenditure^33^. We, however, did not observe significant change in the circulating concentration of these hormones that could be associated with the elevation of BT in POMCβ-KO mice (Supplementary Figure 3F). Recent evidence implies that leptin exerts potential pyrexic effects in rodents by limiting heat dissipation^34–37^. We therefore tested whether altered leptin signaling could contribute to the observed increase in core BT. To do this, recombinant leptin was administered, twice daily, over a period of 3 days and core BT followed by telemetry. Of note, the appetite-suppressing effects of leptin were identical between mouse genotypes, as evidenced by similar reductions of food intake and body mass in leptin-treated versus NaCl-treated animals (Supplementary Figure 4A). This, together with the unaltered stimulating effect of peripheral ghrelin on food intake (Supplementary Figure 4B), indicates that the hypothalamic circuitry controlling feeding is intact in the absence of PGC-1β in POMC neurons. In stark contrast, chronic administration of leptin in particular during the early daytime—when endogenous leptin levels are low—led to a significant increase of core BT levels in POMCβ-KOs, but not in control (CTRL) littermates (Fig. 4A). This suggests that leptin is more potent in altering thermoregulation in POMCβ-KO mice than in CTRL animals. Similar discrepancies regarding the putative pyrexic effect of leptin were observed in other mouse lines and models (reviewed in^36^). However, contrary to ob/ob mice^34^, leptin administration did not significantly affect tail heat loss in CTRL or POMCβ-KO animals either during the day or night in our study, while nighttime tail temperature was significantly reduced regardless of treatment in the POMCβ-KO animals, suggesting a decreased heat dissipation in this context that could be linked with increased BT (Fig. 4B,C).

Lastly, we investigated the effect of a single acute leptin injection on gene expression in the ARC of 24-h fasted CTRL and POMCβ-KO mice (Fig. 4D–F, Supplementary Figure 4C). Our results show that the expression of some of the key transcripts associated with leptin signaling, namely *Protein tyrosine phosphatase non-receptor type 1* (*Ptpn1*; also called *Ptp1b*), *Ptpn11* (also called *Src-homology 2 domain containing phosphatase 2*; *Shp2*), and the *Signal transducer and activator of transcription 3* (*Stat3*) were significantly up-regulated by a single leptin injection in the ARC of POMCβ-KO mice, but not in controls (Fig. 4E, Supplementary Figure 4C). Moreover, we also found altered expression of genes involved in cellular metabolism and mitochondrial function (e.g., *Pyruvate dehydrogenase kinase 4* [*Pdk4*]; *Pgc1-α*) in the ARC of POMCβ-KO mice in response to leptin (Fig. 4F). Notably, the expression of the above genes was virtually unchanged in the hippocampus—which also contains POMC neurons^38^—of POMCβ-KO animals (Supplementary Figure 4D). Overall, these results indicate that the absence of PGC-1β in POMC neurons modulates and/or sensitizes these cells in regard to leptin action, which could be associated with the change in BT observed in POMCβ-KO mice.

## Discussion

The control of body temperature in mammals is a multi-layered process influenced both by behavior and physiology^39^. While specific hypothalamic nuclei have been identified as key regulators of thermogenesis^14^, the identity of the cellular mediators and neuronal populations influencing thermoregulation remains incompletely understood. Here, we provide evidence that the transcriptional co-regulator PGC-1β within POMC neurons of the arcuate nucleus is an important part of a neural circuit controlling core temperature in the mouse.

In contrast to observations in global PGC-1β KO mice^22,23^, the specific deletion of PGC-1β in POMC neurons did not alter overall behavior and energy homeostasis. Similarly, POMCβ-KO mice display unchanged physiological responses to acute cold and norepinephrine exposure. Intriguingly however, POMCβ-KO mice show a consistent and reproducible elevation of core BT levels under sub-thermoneutral conditions, which is not linked to changes in the metabolic rate or the expression of thermogenic, metabolic or beigeing genes in BAT or WAT. Importantly, the higher core temperature is restricted to the active/feeding period, as evidenced by the absence of difference between genotypes in daytime core temperature values when mice mostly sleep and barely eat. Supporting a role in food-linked thermoregulation, nighttime fasting normalizes BT values of POMCβ-KO mice to those of their CTRL littermates, while refeeding restored the differences. Importantly, selective ablation of the PGC-1β gene in AgRP neurons did not affect 24-h BT levels, demonstrating a specific involvement of PGC-1β in POMC cells in the regulation of BT.

Leptin is an adipose-derived hormone essential for the control of food intake and energy balance^14^. In the ARC, leptin activates and inhibits the activity of POMC and NPY/AgRP neurons, respectively, and thereby lowers appetite and elevates energy expenditure^40^. Of note, the action of leptin on food intake is not compromised in POMCβ-KO mice, thus dissociating the role of PGC-1β on leptin action from appetite control. The significance of leptin action in the context of thermoregulation is much less clear, as studies have reported thermogenic (i.e., increasing BAT activity), vasoconstrictor, and vasodilator effects of exogenous leptin (reviewed in Ref.^36^). Recent evidence, however, suggests that leptin is rather pyrexic than thermogenic. In this context, leptin induces rapid changes to defend against drops in BT by reducing heat loss (e.g., by increasing vasoconstriction) without altering energy expenditure and behavioral regulation of BT^36^. In our study, leptin-injected CTRL animals display an elevation of their overall 24-h BT levels, in line with previous findings^41–43^. However, we only observed a significant effect of exogenous leptin on daytime BT—when endogenous leptin levels are low—in POMCβ-KO animals. This, together with their increase nighttime core BT in the absence of change in endogenous leptin levels, indicate that leptin is more potent in altering thermoregulatory mechanisms in the absence of PGC-1β expression in POMC neurons in the active, feeding phase.

A potential modulation of the thermoregulatory effects of leptin by PGC-1β in POMC neurons was furthermore implied by the specific changes in some, but not all of the leptin-regulated gene expression in the ARC of POMCβ-KO mice. For example, the induction of *Pdk4* by leptin was potentiated in the absence of PGC-1β. Moreover, genes encoding proteins involved in leptin signaling were at least in part induced in the POMCβ-KO mice, e.g. *Ptpn1* or *Ptpn11*, which could point towards a sensitization of this pathway. Interestingly, partial deletion of *Ptpn11* in the mouse hypothalamus and forebrain cortex impairs the regulation of BT and energy balance at both ambient and thermoneutral temperatures^44^. The selectivity of the effect of PGC-1β on leptin action was observed in the blunted effect of leptin on other genes, e.g. the reduction of the expression of PGC-1α, whose expression is negatively regulated by leptin in the ARC^45^. Finally, this selectivity of pathway modulation is substantiated by the ability of leptin to normalize the aberrant gene expression caused by the POMC neuron-specific ablation of PGC-1β, e.g. that of *Acaca*. Future work using e.g., fluorescence-assisted cell sorting combined to transcriptional or proteome profiling of POMC neurons could shed light on the regulation of PGC-1β and downstream targets in this specific cell population in response to leptin. This is especially important as several transcriptionally and functionally distinct subsets of POMC neurons have been described in the ARC^46^. Moreover, besides non-POMC neurons, other cell populations within the ARC-median eminence complex, including mural cells and tanycytes, express *Ppargc1b*^46^, which could account for the unchanged *Ppargc1b* expression observed in our study using a conventional approach to isolate the ARC^16^.

Using infrared thermography, we observed that the temperature at the tail—a main effector of heat dissipation in the mouse^47^—of POMCβ-KO animals was reduced at night. However, contrary to the ob/ob mouse model^34^, leptin treatment did not further boost this effect in either the CTRL or the POMCβ-KO in this study. Of note, ob/ob mice display a dramatic change in BT at baseline and in response to leptin administration, while POMCβ-KO mice only show a mild change in these conditions. Thus, any modulation of tail vasoconstriction by peripheral, exogenous leptin might be too subtle to be detected in the CTRL or POMCβ-KO mice with our methods. Second, as in the alteration of overall BT, the sensitization of POMCβ-KO animals to endogenous leptin during nighttime might mask any additional effect of exogenous leptin in regard to heat dissipation in the tail. Alternatively, other mechanisms of heat retention (e.g., change in skin properties^48^) could contribute to limit heat loss and defend BT in POMCβ-KOs, potentially also mediated by other hormones such as thyroid hormone, which plays a key role in vascular heat conservation and dissipation processes^49,50^. Moreover, PGC-1β regulates a broad gene program in peripheral tissues, particularly in response to dietary-fat intake and thyroid hormone (reviewed in^18^).

Our study also cannot fully exclude the involvement of central efferent mechanisms and/or altered skeletal muscle function that could lead to increased muscle-based thermogenesis^51–53^. Likewise, we cannot rule out the possibility that other neuronal populations, innervated by POMC cells and/or sensitive to leptin, could contribute to the elevation of the BT of POMCβ-KOs. For instance, the ARC sends neuronal projections to the dorsomedial hypothalamus^54^, which contains a distinct population of prolactin-releasing peptide neurons, expressing the leptin receptor, and involved in the thermoregulatory effect of leptin^43^. Moreover, release of the POMC-derived α-melanocyte-stimulating hormone (α-MSH) from the ARC to the medial preoptic area can influence the daily amplitude of BT^55^. Finally, potential pitfalls when using the POMC-Cre mouse model e.g., the scattered Cre activity in other areas of the brain, should be acknowledged^56,57^. Hence, it would be essential to evaluate whether and how PGC-1β-expressing POMC neurons communicate with intra- and extra-ARC cells to modulate core BT, particularly in response to leptin.

## Conclusion

**Figure 5:**
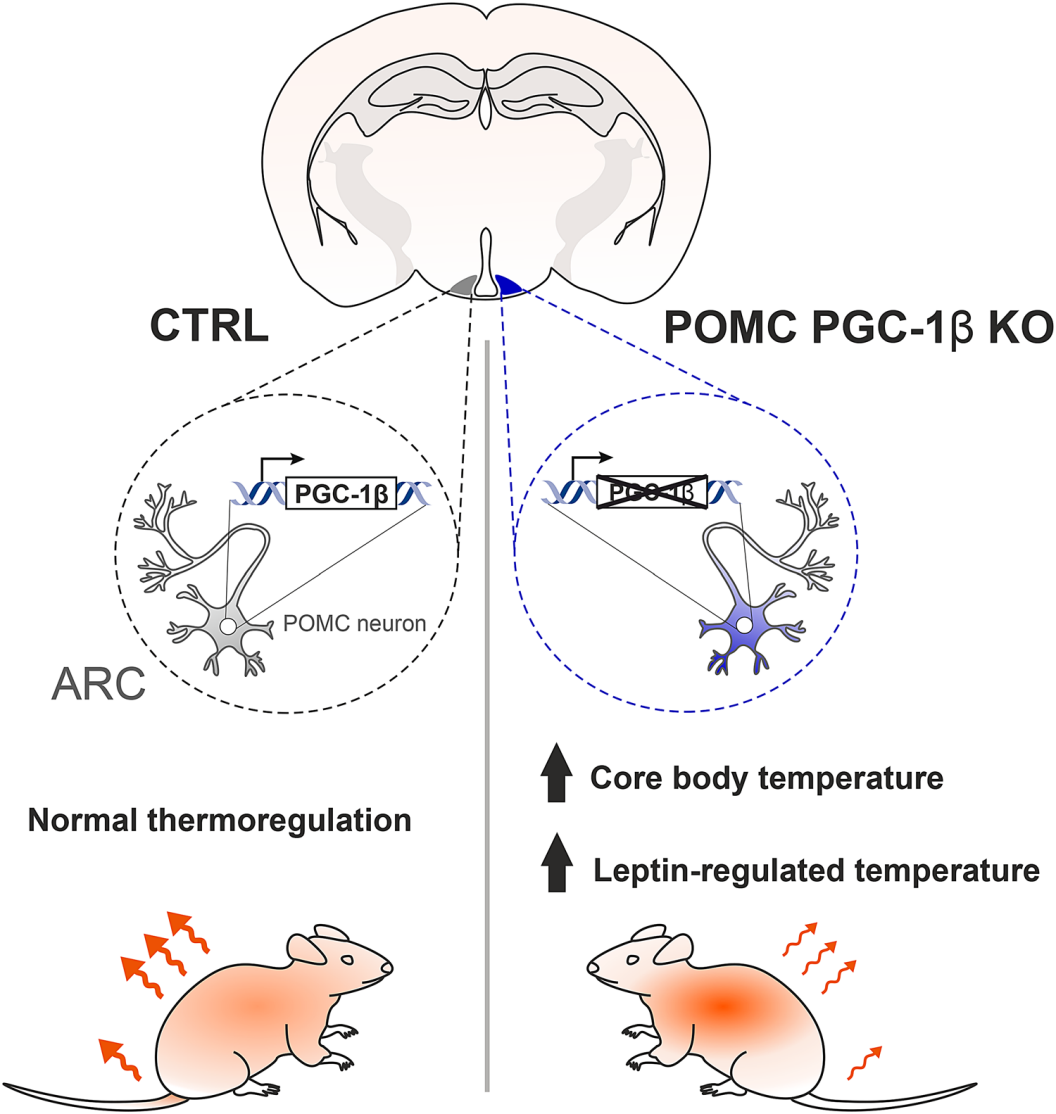
The effect of POMC-expressing neuronal PGC-1β deletion on core body temperature in the mouse. PGC-1β in POMC-positive neurons of the arcuate nucleus affects postprandial thermoregulation, at least in part by modulating leptin signaling in these cells.

Our findings uncover a role for PGC-1β in POMC neurons in the thermoregulation in the mouse (Fig. 5). Given the importance of the PGC-1 coactivators in mitochondrial function^19,58^, the conserved relation between mitochondrial dynamics and the formation and maintenance of neuronal connections^15,59^, together with the trophic action of leptin on hypothalamic cells^60^, future research should evaluate whether the arcuate neuronal circuitry is functionally and structurally affected upon PGC-1β deletion in POMC cells.

## Methods

### Animals

AgRP^Cre/+^ (Agrp^*tm*1*(cre)Lowl*^; #012899), POMC^Cre/+^ (STOCK tg(Pomc1-cre) 16Lowl/J; #005965) and *Ppargc1b*^*flox/flox*^ mice (B6;129X1-Ppargc1btm1Dpk/J; #012388) were obtained from the Jackson Laboratory (stock no. 006149; Bar Harbor, USA). Genotyping and testing for unexpected recombination events was performed as previously described^16^. All mice were maintained on a C57BL/6J genetic background in the animal facility of the Biozentrum (University of Basel) under a 12:12 h light/dark cycle (lights on at 6:00 am). *Ppargc1b*^*flox/flox*^ mice were used as littermate controls (CTRL) in our study. Unless specifically mentioned, all experiments were performed in young adult male mice (3- to 6-mo-old) housed in standard cages at 23 °C with bedding substrates and free access to regular chow diet (#3432, KLIBA NAFAG) and water. All experiments were performed in accordance with the principles of the Basel Declaration and with Federal and Cantonal Laws regulating the care and use of experimental animals in Switzerland, as well as institutional guidelines of the Biozentrum and the University of Basel. The protocol with all methods described here was approved by the “Kantonales Veterinäramt” of the Kanton Basel-Stadt, under consideration of the well-being of the animals and the 3R principle.

### Body composition analysis

Body composition was assessed with an EchoMRI‐100 analyzer (EchoMRI Medical Systems). Fat and lean mass were normalized to total body mass.

### Body temperature and locomotor activity recordings

General locomotor activity and core body temperature data were acquired with the E-Mitter Telemetry System (Starr Life Sciences) from single-caged animals placed in an environment-controlled cabinet (UniProtect Air Flow Cabinet, Bioscape) as previously described^61^.

### Infrared thermography

Mouse tail base temperature was measured at 23 °C room temperature using an infrared camera (FLIR T530). Animals were subjected to i.p. injections, twice daily, of NaCl and recombinant leptin (as described below). Three hours post-injection, animals were placed on a cage lid, allowed to move freely and a minimum of two infrared pictures were taken from the same animal within one minute, either during the dark phase under red light or during the light phase under white light. The ResearchIR thermal analysis software (FLIR) was used to draw a Region of Interest (ROI of 3*3 block of pixels; Fig. 4B) at the base of the tail. Factory settings were used to convert raw pixel counts to units of temperature and only pictures taken from the same angle were included in the analysis.

### Comprehensive laboratory animal monitoring system

A comprehensive animal metabolic monitoring system (CLAMS; Columbus Instruments) was used to evaluate food consumption, oxygen consumption (VO_2_) and CO_2_ production (VCO_2_) from individually housed mice within an environment-controlled cabinet with a temperature set at 23 °C. Food intake was measured with a scale of the center feeder. We studied 8 mice per genotype in both basal fed and 24-h fasted/refed conditions, after an acclimatization of 2 days.

### Cold exposure and norepinephrine tests

For cold exposure challenge, animals were individually placed and acclimatized to CLAMS cages in early daytime with free access to water but no food for at least 6 h at 23 °C. Five days later, animals were placed in pre-cooled CLAMS cages without food access for 6 h at 4 °C. Core body temperature measurements were obtained using Anipill Temperature Implant (Phymep). For noradrenaline administration experiments, mice were individually housed at thermoneutrality (29–30 °C) for three weeks. Thermoneutrality-acclimatized mice were rapidly anesthetized during early daytime using pre-warmed pentobarbital (Esconarkon; 90 mg/kg) and immediately placed in pre-warmed CLAMS cages sitting in a 30 °C cabinet. Basal oxygen consumption was measured for ca. 30 min and then mice were given a sub-cutaneous injection of 1 mg/kg of L-( −)-Norepinephrine ( +)-bitartrate salt monohydrate (Sigma). Oxygen-consumption rates were recorded for an additional 90 min, before recovery from the anesthesia.

### Ghrelin and leptin sensitivity tests

To acclimatize the mice for these tests, animals were mock-handled on several days prior to the experiments. Intraperitoneal injections were performed with 2 and 5 mg/kg body weight of rat ghrelin (Bachem H-4862) and rat leptin (R&D 498-OB-05M), respectively, diluted in NaCl 0.9%. Food pellets were weighed and exchanged after each injection. Note that vehicle control (NaCl), ghrelin (Ghr) and leptin (Lep), were injected in subsequent experiments into the same animals. Ghrelin injection was performed at 12 pm and food intake was measured 1, 2 and 3 h after injection. For chronic leptin treatment, leptin was injected twice daily (i.e., at 7 am and 5 pm) over 3 days. Food intake and body weight were measured at the time of injections. For acute leptin injection, mice were fasted for 24 h (from 6 am to 6 pm) and a single leptin injection (same dose as above) was performed at 6 pm.

### Tissue collection

Animals were sacrificed by CO_2_ asphyxiation either during early daytime or early nighttime. Blood was rapidly collected from the heart and plasma was further isolated. Adipose tissues (i.e., BAT and iWAT) were quickly dissected and flash-frozen. Brain tissue was quickly sampled and regions comprising the hypothalamus (centered around the arcuate nucleus and median eminence, see Supplementary Figure 1A), cortex and hippocampus were dissected as previously described^62^.

### Blood parameters analysis

Daytime plasma parameters (e.g., glucose and triglyceride levels) were measured with a COBAS c111 analyzer (Roche Diagnostics). Nighttime plasma insulin and leptin were determined with ELISA kits (Crystal Chem).

### DNA/RNA extraction and real-time qPCR

DNA was isolated and purified as previously described^16^. Total RNA was extracted from brain and adipose tissues using TRI-Reagent (Sigma). RNA quantity and purity were evaluated using the NanoDrop OneC spectrophotometer (Thermo Scientific; A260/A280 and A260/A230 values were > 1.7). cDNAs were synthesized from 300 ng^−1^ μg of total RNA using the High Capacity cDNA RT Kit (Applied Biosystems). Quantitative real-time PCR was performed with Fast SYBR Green using the StepOnePlus Real-Time PCR System (Applied Biosystems). PCR reactions were done in duplicate with the addition of negative controls (i.e., no reverse transcription and no template controls). Relative expression levels were determined using the comparative ΔΔCT method to normalize target gene mRNA to *Hprt* (brain tissues) and *Actb* (adipose tissues). Primers were designed and tested as previously described^63^. Primer sequences are listed in Supplementary Table 1.

### Statistical analysis

No statistical methods were used to predetermine sample size. The n number used per genotype and the statistical test used for each experiment is indicated in the Fig. legend. Data are expressed as mean ± SEM and were represented and analyzed with the GraphPad Prism 7.0 software. Comparisons between two groups were performed with unpaired Student’s *t* test. For assessment between two independent variables, two-way (repeated measures; RM) ANOVA was used followed, when appropriate, by Tukey’s or Sidak’s multiple comparisons test for interaction and main effects, respectively. For complex datasets with more than two groups and variables (i.e., Fig. 4A—*right panel*), specific pairwise comparisons were defined as of interest prior to data analysis. A value of *P* < 0.05 was considered statistically significant. Symbols used to indicate the different degrees of statistical significance are as follow: *P < 0.05, **P < 0.01, and ***P < 0.001 between mouse genotypes and ^#^*P* < 0.05, ^##^*P* < 0.01, ^###^*P* < 0.001 between experimental conditions.

## Supplementary information

Supplementary file1.

## Abbreviations

AgRP: Agouti-related peptide
ARC: Arcuate nucleus
BAT: Brown adipose tissue
BT: Body temperature
CRTL: Control
Cx: Cortex
HIPP: Hippocampus
POMC: Pro-opiomelanocortin
Ppargc1b/PGC1-β: Peroxisome proliferator-activated receptor gamma coactivator 1β
WAT: White adipose tissue
