# Supplementary material for: PGC-1β-expressing POMC neurons mediate the effect of leptin on thermoregulation in the mouse
Source: Sci Rep. 2020 Oct 15;10:16888. doi: 10.1038/s41598-020-73794-7 (PMC7567876; doi:10.1038/s41598-020-73794-7)
Supplement: Supplementary file 1 — Supplementary file1. [file 41598_2020_73794_MOESM1_ESM.pdf]

Supplementary Information for

PGC-1β-expressing POMC neurons mediate the effect of leptin on thermoregulation in the mouse

Julien Delezie, Jonathan F. Gill, Gesa Santos, Bettina Karrer-Cardel and Christoph Handschin*

Biozentrum, University of Basel, CH-4056 Basel, Switzerland

**Correspondence to:* Christoph Handschin, Biozentrum, University of Basel, Klingelbergstrasse 50/70, CH-4056 Basel, Switzerland, phone: +41 61 207 2378, fax: +41 61 207 2208, email: christoph.handschin@unibas.ch

This PDF file includes:

Figs. S1 to S4
Table S1

Supplementary Figures

Supplemental Figure 1

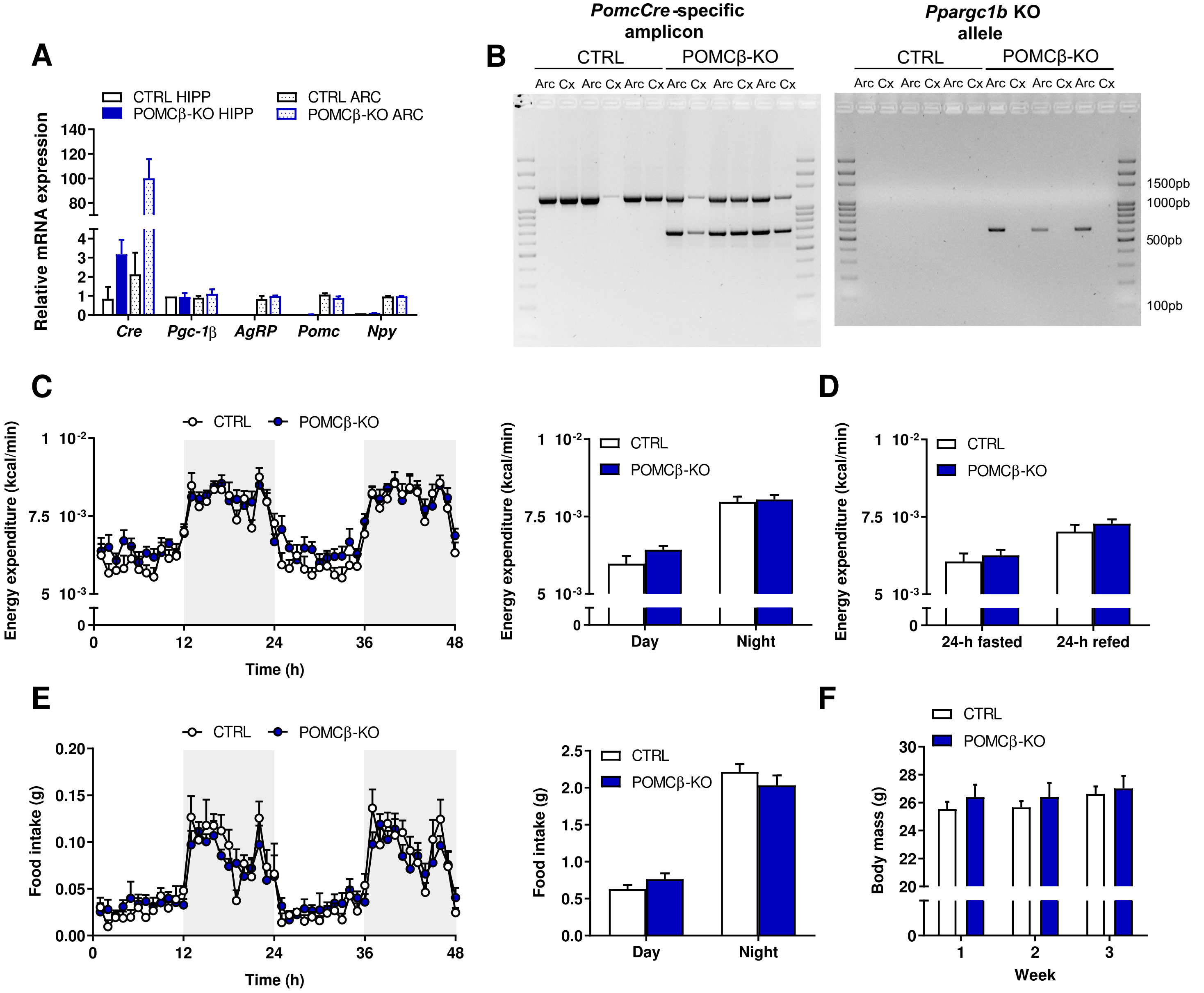

Supplementary Fig. 1. KO validation and energy homeostasis of POMCβ-KO mice.

(**A**) Relative mRNA expression in the hippocampus (HIPP) and arcuate nucleus (ARC) of CTRL and POMCβ-KO mice (n = 3-4 per genotype). Expression values were determined by qPCR and normalized to *Hprt*. Note the enrichment for *AgRP*, *Pomc* and *Npy* transcripts in the ARC. (**B**) PCR detection of Pomc-driven Cre expression and *Ppargc1b* floxed allele recombination in the arcuate nucleus (Arc) and cortex (Cx) brain regions. Note that gel images do not originate from the same gel and that we have used *Ppargc1b* floxed animals as CTRL in our study. Accordingly, there is no detection of the transgene in CTRL brain regions and only a successful Cre-mediated recombination of the *Ppargc1b* floxed allele in the hypothalamus of POMCβ-KO (i.e., $\textit{Ppargc1b}^{flox/flox}$;$\textit{POMC}^{Cre/+}$) mice. (**C**) Energy expenditure under basal fed conditions and (**D**) 24-h fasted-refed conditions in relation to Fig. 1E, F (n = 7-8 per genotype). (**E**) Food intake under basal fed conditions as evaluated with the CLAMS (n = 7-8 per genotype). (**F**) Body mass of single-caged implanted mice housed in an environment-controlled cabinet under basal fed conditions in relation to Fig. 2A-D (n = 7-8 per genotype). Results are expressed as mean ± SEM.

Supplemental Figure 2

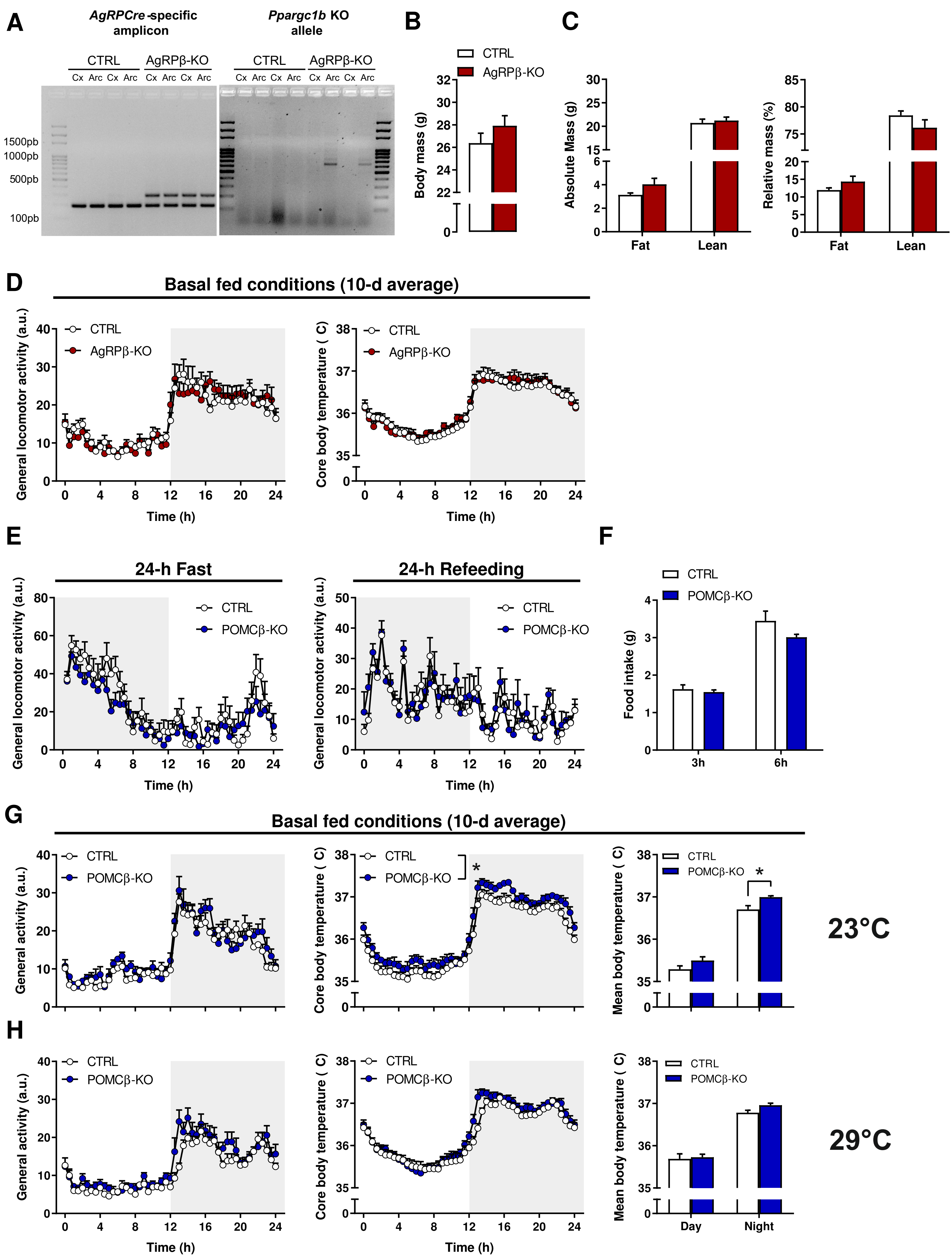

Supplementary Fig. 2. KO validation of AgRPβ-KO mice and telemetry data from both AgRPβ-KO and POMCβ-KO mice.

(**A**) PCR detection of AgRP-driven Cre expression and *Ppargc1b* allele recombination in the arcuate nucleus (Arc) and cortex (Cx) brain regions. (**B**) Daily food intake, (**C**) body mass and absolute and normalized fat and lean mass of 4-month-old CTRL and AgRPβ-KO mice (non-implanted and group-housed). (**D**) Daily gross locomotor activity (*left*) and core body temperature levels (*right*) of 3 to 4-month-old CTRL and AgRPβ-KO mice in basal fed conditions as measured by telemetry over a 10-d period. (**E**) Gross locomotor activity during a 24-h fasting period (*left*) and during a 24-h refeeding period (*right*) of CTRL and POMCβ-KO mice in relation to Fig. 2C,D (n = 7-8 per genotype). (**F**) Food intake 3 and 6 h after refeeding as evaluated with the CLAMS (n = 7-8 per genotype). (**G-H**) Daily gross locomotor activity (*left*) and core body temperature levels (*middle, right*) of 4 to 5-month-old CTRL and POMCβ-KO mice in basal fed conditions as measured by telemetry over a 10-d period. Note that this specific mouse cohort was first housed at 23°C (**G panels**) before subsequent housing at 29°C (**H panels**). Results are expressed as mean ± SEM.

Supplemental Figure 3

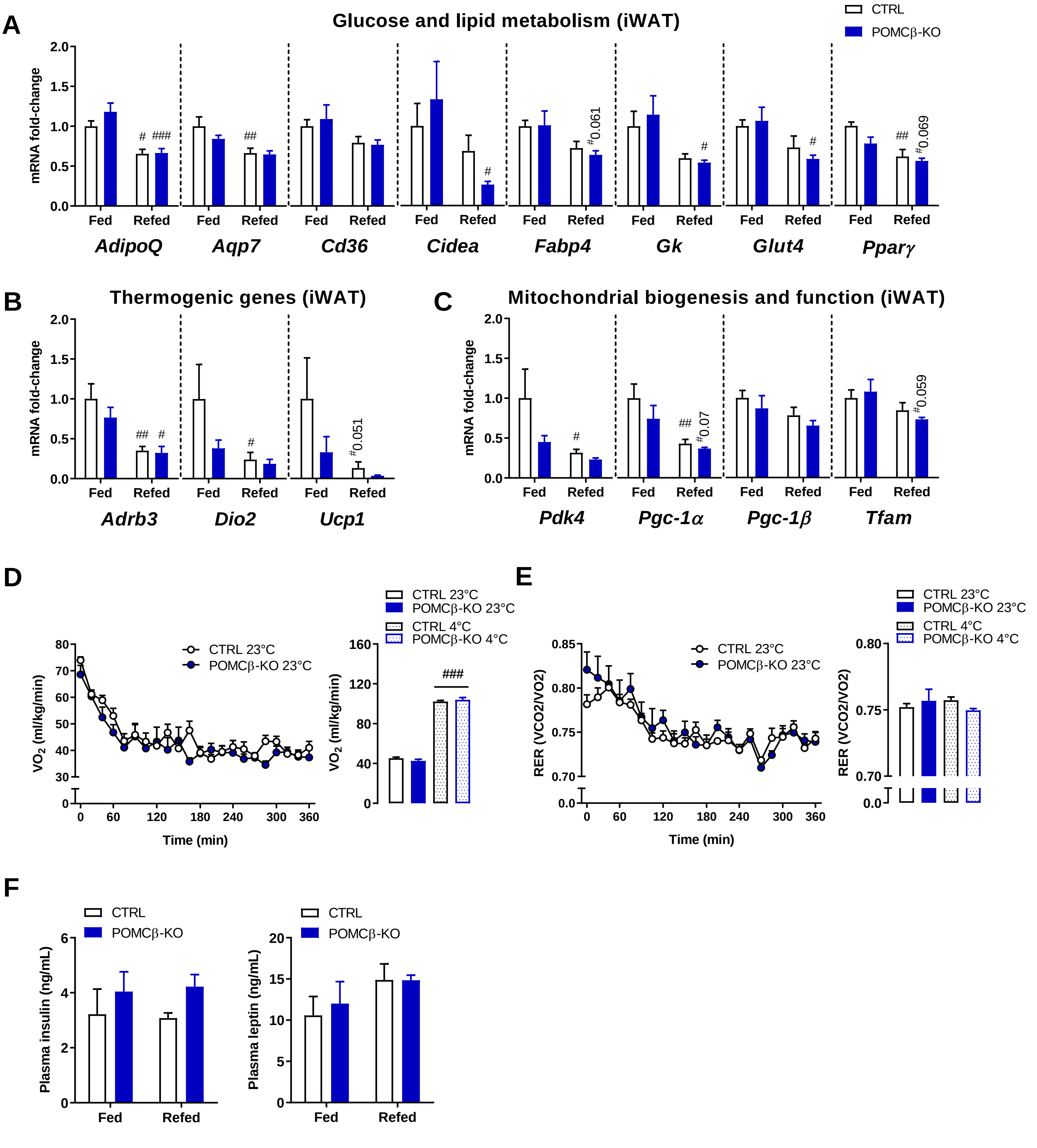

Supplementary Fig. 3. Loss of POMC-PGC-1β does not alter WAT gene expression, plasma insulin and leptin levels.

(**A-C**) Gene expression in the inguinal white adipose tissue (WAT) of CTRL and POMCβ-KO mice. Expression values were determined by qPCR and normalized to *Actb*. Data is shown as the average fold-change ± SEM relative to the expression in CTRL set to 1 (n = 4-5 per genotype). (**D**) O_2 consumption and (**E**) respiratory exchange ratio (RER) values during acute cold exposure (n = 7-8 per genotype). (**F**) Early nighttime plasma insulin and leptin levels in both fed and refed conditions (n = 4-5 per genotype). $^{\#}$ indicates a significant difference between experimental conditions ($^{\#}$ $P < 0.05$; $^{\#\#}$ $P < 0.01$; $^{\#\#\#}$ $P < 0.001$). Two-way ANOVA (a,b,c).

Supplemental Figure 4

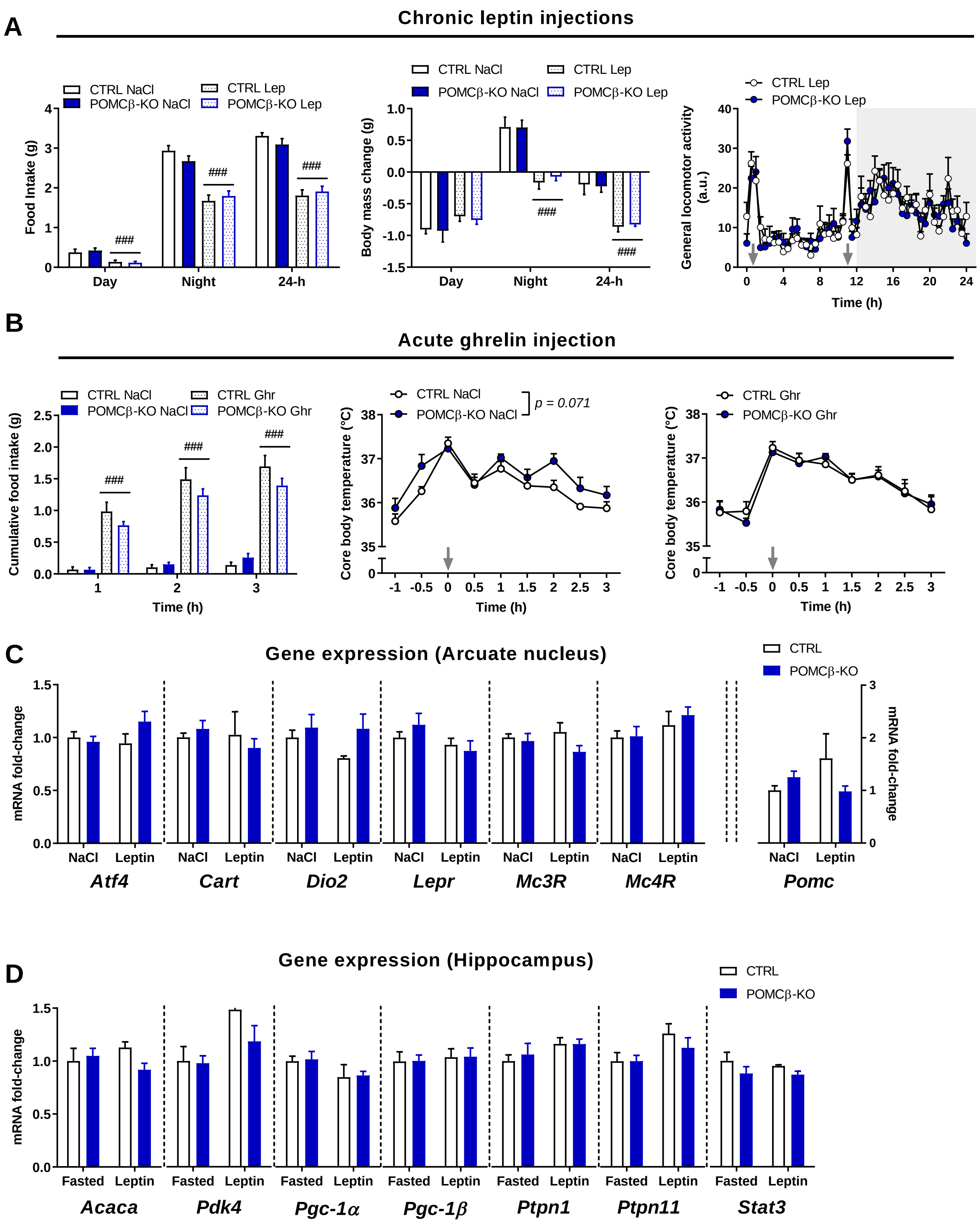

Supplementary Fig. 4. Physiological responses to chronic leptin and acute ghrelin injections of POMCβ-KO mice.

(**A**) Change in food intake (*left),* body mass (*middle*) and gross locomotor activity (*right panel*) of CTRL and POMCβ-KO mice in response to twice daily i.p. injections of NaCl vs. leptin in relation to Fig. 4A. Gray arrows indicate the time of injection (n = 7-8 per genotype). (**B**) Change in food intake (*left),* core body temperature (*middle and right panels*) of CTRL and POMCβ-KO mice in response to a single acute injection of NaCl vs. ghrelin (Ghr). The gray arrows indicate the time of injection (n = 7-8 per genotype). (**C**) Gene expression in the arcuate nucleus and (**D**) hippocampus of CTRL and POMCβ-KO mice. Expression values were determined by qPCR and normalized to *Hprt*. Data is shown as the average fold-change ± SEM relative to the expression in CTRL (n = 4-7 per genotype per condition). $^{\#}$ indicates a significant difference between experimental conditions ($^{\#}$ $P < 0.05$; $^{\#\#}$ $P < 0.01$; $^{\#\#\#}$ $P < 0.001$). Two-way ANOVA (a,b).

Supplementary Table 1. Primer pairs used in qPCR

Gene name	Forward	Reverse
Acaca	GCCTCTTCCTGACAAACGAG	TGACTGCCGAAACATCTCTG
AdipoQ	TGTTCCTCTTAATCCTGCCCA	CCAACCTGCACAAGTTCCCTT
Adrb3	CCTTCAACCCGGTCATCTAC	AGCCATCAAACCTGTTGAGC
Agrp	GGCCTCAAGAAGACAACTGC	GCAAAAGGCATTGAAGAAGC
Aqp7	AATATGGTGCGAGAGTTTCTGG	ACCCAAGTTGACACCGAGATA
Atf4	GGGTTCTGTCTTCCACTCCA	AAGCAGCAGAGTCAGGCTTTC
Cart	CGAGAAGAAGTACGGCCAAG	GGAATATGGGAACCGAAGGT
Cd36	AAGAGGTCCTTACACATACAGAG TTC	AGCTGCTACAGCCAGATTCA
Cidea	GCAGCCTGCAGGAACTTATC	CCATTTCTGTCCCTTTTCCA
Cpt1a	TACTACGCCATGGAGATGCT	TGACGTGTTGGATGGTGTCT
Cpt1b	GTCGCTTCTTCAAGGTCTGG	AAGAAAGCAGCACGTTCGAT
Dio2	CAGTGTGGTGCACGTCTCCAATC	TGAACCAAAGTTGACCACCAG
Esrra	CAAGAGCATCCCAGGCTT	GCACTTCCATCCACACACTC
Esrrb	GGGAGCTTGTGTTCCTCATC	ATCTCCATCCAGGCACTCTG
Esrrg	CCCAAGAGACTGTGCTTAGTG	GGGCAGCTGTACTCTATGTTAC
Fabp4	TTTCCTTCAAACTGGGCGTG	CATTCCACCACCAGCTTGTC
Foxo1	ACATTTCGTCCTCGAACCAGCTCA	ATTTCAGACAGACTGGGCAGC GTA
Gk	ATCCGCTGGCTAAGAGACAACC	TGCACTGGGCTCCCAATAAGG
Glut4	GATTCTGCTGCCCTTCTGTC	ATTGGACGCTCTCTCTCCAA
Lepr	CGTGGTGAAGCATCGTACTG	GGGCCATGAGAAGGTAAGGT
Mc3r	TCAAACGCGAAGAAAGTGCA	AGACAACGCACTTACCAGGA
Mc4r	GGACTCTGAAAAGACCCCGA	TGCCTGTAGAAGTTGGGAGG
Npy	TCGCTCTATCTCTGCTCGTG	AATCAGTGTCTCAGGGCTGG
Pdk4	CCGCTGTCCATGAAGCA	GCAGAAAAGCAAAGGACGTT
Pomc	CATTAGGCTTGGAGCAGGTC	TCTTGATGATGGCGTTCTTG
Pparg	TGTGGGGATAAAGCATCAGGC	CCGGCAGTTAAGATCACACCTA T
Ppargc1a	AGCCGTGACCACTGACAACGAG	GCTGCATGGTTCTGAGTGCTAA G
Ppargc1b (Ex 5)	ATGCTTCCCTCACACCTCAG	GCTTTTGCCTTGTAGGCTTG
Ptpn1	CCGAGATGTCAGCCCTTTTG	CCACACCATCTCCCAGAAGT
Ptpn11	GAAACGGTCATTCAGCCACT	GCAGCCAAGGAGTCATCTTC
Ptpn2	AGAGTGGCCAAGTTTCCAGA	CACACCATGAGCCAGAAATG
Socs3	GCGGGCACCTTTCTTATCC	TCCCCGACTGGGTCTTGAC
Stat3	TGGCACCTTGGATTGAGAGT	CGATCCGGGCAATTTCCATT
Tfam	GGTCGCATCCCCTCGTCTA	GGATAGCTACCCATGCTGGAA A
Ucp1	AGGCTTCCAGTACCATTAGGT	CTGAGTGAGGCAAAGCTGATTT
